# TLR expression profile of human gingival margin-derived stem progenitor cells

**DOI:** 10.4317/medoral.20593

**Published:** 2015-11-30

**Authors:** Karim Fawzy-El-Sayed, Mohamed Mekhemar, Sabine Adam-Klages, Dietrich Kabelitz, Christof Dörfer

**Affiliations:** 1 Clinic for Conservative Dentistry and Periodontology, School of Dental Medicine, Christian Albrechts University, Kiel, Germany; 2 Oral Medicine and Periodontology Department, Faculty of Oral and Dental Medicine, Cairo University, Egypt; 3 Universitätsklinikum Schleswig Holstein, Institut für Immunologie, Kiel, Germany; Clinic for Applied Cellular Therapy, Christian Albrechts University, Kiel, Germany

## Abstract

**Background:**

Gingival margin-derived stem/progenitor cells (G-MSCs) show remarkable periodontal regenerative potential *in vivo*. During regeneration, G-MSCs may interact with their inflammatory environment via toll-like-receptors (TLRs). The present study aimed to depict the G-MSCs TLRs expression profile.

**Material and Methods:**

Cells were isolated from free gingival margins, STRO-1-immunomagnetically sorted and seeded to obtain single colony forming units (CFUs). G-MSCs were characterized for CD14, CD34, CD45, CD73, CD90, CD105, CD146 and STRO-1 expression, and for multilineage differentiation potential. Following G-MSCs’ incubation in basic or inflammatory medium (IL-1β, IFN-γ, IFN-α, TNF-α) a TLR expression profile was generated.

**Results:**

G-MSCs showed all stem/progenitor cells’ characteristics. In basic medium G-MSCs expressed TLRs 1, 2, 3, 4, 5, 6, 7, and 10. The inflammatory medium significantly up-regulated TLRs 1, 2, 4, 5, 7 and 10 and diminished TLR 6 (*p*≤0.05, Wilcoxon-Signed-Ranks-Test).

**Conclusions:**

The current study describes for the first time the distinctive TLRs expression profile of G-MSCs under uninflamed and inflamed conditions.

**Key words:**Stem cells, TLR, gingiva, polymerase chain reaction, FACS.

## Introduction

The human gingiva covers the tooth-bearing alveolar bone with its inserting teeth. One of its distinguished features is its significant regenerative and wound healing capacity with little if any evidence of scarring (1). The numerous functions of gingival connective tissue fibroblasts, their broad spectrum in responsiveness to growth/differentiation factors as well as in the capacity to produce an array of specific extra cellular matrix proteins during healing demonstrates their heterogeneous nature (2-6) and implies the presence of a population of stem/progenitor cells, giving rise to these heterogeneous cells. Earlier studies described the isolation of stem/progenitor cells from oral soft tissues (7-12). Current investigations reported promising characteristics of gingival stem/progenitor cells, including its immunomodulatory properties (13) as well as their compatibility with alginate hydrogel microbeads scaffolds (14). In a recent study, free gingival margin-derived stem/progenitor cells (G-MSCs) demonstrated a remarkable regenerative capacity *in vivo* (15).

Toll-like receptors (TLRs), major molecules linking the innate and adaptive immunity, are germ line-encoded pattern-recognition receptors (PRRs) detecting specific pathogen-associated molecular patterns (PAMPs), thereby promoting immune cells’ activation (16,17). They function as sensors for invading pathogens and are involved in autoimmune, chronic inflammatory and infectious diseases’ pathogenesis (18). To date, 10 functional human TLRs have been characterized (19). Depending on their cellular localization and their PAMP ligands, TLRs are divided into extracellular and intracellular ones. The first group is expressed on the cell surface and they mostly identify microbial membrane constituents including lipids and lipoproteins (TLR1, TLR2, and TLR6), lipopolysaccharide (LPS) (TLR4), and flagellin (TLR5). The second group is expressed intracellularly, where they identify double-stranded RNA (TLR3), single-stranded viral RNA (TLR7 and TLR8) and unmethylated CpG DNA of viruses and bacteria (TLR9) (20).

Multipotent stromal cells (MSCs) of different origin have been shown to express functional TLRs in specific patterns, making them selectively sensitive to microbial compounds. When triggered TLRs can modulate MSCs’ proliferative, immunosuppressive, migratory and differentiation potentials (19,21-23). Differential expressions of TLRs 1, 2, 3, 4, 5, 6, were described on human and mural adipose and bone marrow derived MSCs, on human umbilical cord blood MSCs (UCB-MSCs), on human Wharton Jelly MSCs (WJ-MSCs), and on human MSCs from the dental pulp and the dental follicle (22,24,25). Results showed that the specific pattern of TLRs expression varies according to the MSCs’ tissue of origin, which could have an implication on the MSCs’ therapeutic potential during transplantation in inflammatory environments *in-vivo* (26).

G-MSCs are currently experimentally employed in therapeutic modalities for inflammatory conditions including the treatment of periodontitis (15) and colitis (13). To date no TLRs’ expression profile exists for the G-MSCs. The aim of the present study is to characterize the G-MSCs’ TLR expression profile in uninflamed and inflamed conditions.

## Material and Methods

- Isolation and culture of G-MSCs

G-MSCs isolation was done as previously described (27). Briefly, after obtaining the patients’ informed consent (Ethical Committee IRB- Approval number D 444/10), free gingival collars from five individuals (n=5) were surgically excised at the department of periodontology of the Christian-Albrechts-University-Kiel, Germany. The free gingival tissue collars were detached, de-epithelised, cut into small pieces, rinsed several times with Minimum Essential Medium Eagle Alpha Modification (α-MEM; Sigma-Aldrich GmbH, Hamburg, Germany) supplemented with antibiotics (100 U/ml penicillin, 100 µg/ml streptomycin) and 1% amphotericine (all from Biochrom AG, Berlin, Germany) and placed into dry 75 ml culture flasks (Sarstedt AG, Nümbrecht, Germany) for 30 minutes to adhere to their bottoms. Subsequently, the basic medium consisting of α-MEM, supplemented with 15% fetal calf serum (FCS; HyClone, Logan, UT, USA), 400 mmol/ml L-glutamine (Biochrom), 100 U/ml penicillin, 100 µg/ml streptomycin and 1% amphotericine was carefully added. The flasks were incubated in 5% carbon dioxide at 37°C and cells left to grow out. The culture flasks were periodically checked by phase contrast inverted microscopy and the basic medium changed three times per week. After reaching 80-85% confluence, cells were detached with 0.10% trypsin-EDTA (Biochrom) and counted. Their viability was tested using Trypan Blue (Sigma-Aldrich) to be finally seeded at a density of 30 cells/cm² in 75 ml culture flask in basic medium and the flasks were incubated in 5% carbon dioxide at 37°C. After the first passage cells reached 80-85% confluence, they were subjected to immunomagnetic cell sorting using anti-STRO-1 (BioLegend, San Diego, CA, USA) and anti-IgM MicroBeads (Miltenyi Biotec, Bergisch Gladbach, Germany) antibodies according to the manufacturers’ instructions (MACS; Miltenyi Biotec). The positively sorted cell fractions (G-MSCs) were seeded out to form colony-forming units (CFUs).

- Colony-forming units (CFUs) 

To assess colony-forming efficiency, G-MSCs were cultured in basic medium at a density of 1.63 cells/cm2. Aggregates of 50 or more cells were scored as colonies. On day 12 a representative sample of the cultures were fixed with 4% formalin, stained with 0.1% crystal violet. From the remainder of the CFUs forming G-MSCs single colonies were then detached by cell scrapers (28,29) and seeded in new 75 ml flasks in basic medium.

- Flow cytometric analysis

After reaching confluence, a sample of the G-MSCs were characterized by flow cytometry for the predefined MSCs’ surface marker constellation (30); namely CD14, CD34, CD45, CD73, CD90 and CD105 (all from Becton Dickinson). Binding of the primary antibodies and the corresponding isotype controls was performed according to standard protocols using FcR Blocking Reagent (Miltenyi Biotec) and evaluated with FACSCalibur E6370 and FACSComp 5.1.1 software (Becton Dickinson).

- Multilineage differentiation potential

To test for osteogenic differentiation potential, third passage 2×104 G-MSCs were cultured on 6-well culture plates in osteogenic inductive medium (PromoCell, Heidelberg, Germany). As controls, G-MSCs were cultured in basic medium. At day 14, cell cultures were stained with Alizarin Red (Sigma-Aldrich) (30), to label calcified deposits. To test the adipogenic differentiation potential, third passage 3×105 G-MSCs were cultured on 6-well culture plates in adipogenic inductive medium (PromoCell). As a control G-MSCs were cultured in basic medium. The presence of lipid drops was evaluated by staining with Oil-Red-O (Sigma-Aldrich) (30). To test the chondrogenic differentiation potential, micro-masses of third passage 3×104 G-MSCs were incubated with chondrogenic inductive medium (PromoCell) in 6-well culture plates (Sarstedt AG, Germany). As a control, G-MSCs were cultured in basic medium. Chondrogenic differentiation was evaluated at day 35 by staining of glycosaminoglycans with Alcian Blue (Sigma-Aldrich) (30,31). All media were renewed three times per week.

- Inflammatory medium

To test the effect of the inflammatory environment on the G-MSCs TLR expression profile, a combination of 25 ng/ml IL-1β, 103 U/ml IFN- γ, 50 ng/ml TNF-α, and 3×103 U/ml IFN-α (inflammatory medium, all from PeproTech, Hamburg, Germany) (32) was used. G-MSCs were incubated for 18 hours in the inflammatory medium (G-MSCs-i) as well as basic medium (G-MSCs).

- Flow cytometric determination of TLR expression 

G-MSCs and G-MSCs-i were characterized by flow cytometry for the presence of the different TLRs 1-10 at protein level. For intracellular TLRs staining, cells were fixed, permeabilized with Fix & Perm cell permeabilization kit (Imtec, Antwerpen, Belgium) before incubation. The following antibodies were used: anti-TLR1, anti-TLR3 and anti-TLR9 (all from eBioscience), anti-TLR2, anti-TLR4 and anti-TLR8 (all from Enzo Life Sciences, Lörrach Germany), anti-TLR5 (R&D Systems, Hessen, Germany), anti-TLR6 (BioLegend), anti-TLR7 (Perbio Science, Bonn, Germany) and anti-TLR10 (Acris Antibodies, Herford, Germany). Binding of the primary antibodies and the corresponding isotype controls was performed according to standard protocols using FcR Blocking Reagent (Miltenyi Biotec) and evaluated with FACSCalibur E6370 and FACSComp 5.1.1 software (Becton Dickinson).

- m-RNA Extraction and cDNA Synthesis

m-RNA extraction was performed for G-MSCs and G-MSCs-i using the RNeasy kit (Qiagen, Hilden, Germany) according to manufacturer’s instructions. The obtained RNA was purified using RNase-free-DNase (Promega, Mannheim, Germany), and quantified photometrically. Complementary cDNA was synthesized from 1-13µl of RNA (1 µg/µl) by reverse transcription (RT) using QuantiTect reverse transcription kit (Qiagen) according to the manufacturer’s instructions (Mastercycler gradient; Eppendorf) in a volume of 20 µl reaction mixture containing 4 pmol of each primer, 10µl of the LightCycler Probes Master mixture (Roche Diagnostics) and 5 µl specimen cDNA. Real time polymerase chain reaction (rt-PCR; LightCycler 96 Real-Time PCR System, Roche Molecular Biochemicals, Indianapolis, Indiana, USA) was performed according to the manufacturer’s instructions. Relative quantities of each transcript were normalized according to the expression of PGK1. Primers for TLRs 1 to 10 and the reference gene PGK1 were supplied by Roche and tested on G-MSCs and G-MSCs-i ( Table 1).

**Table 1:**
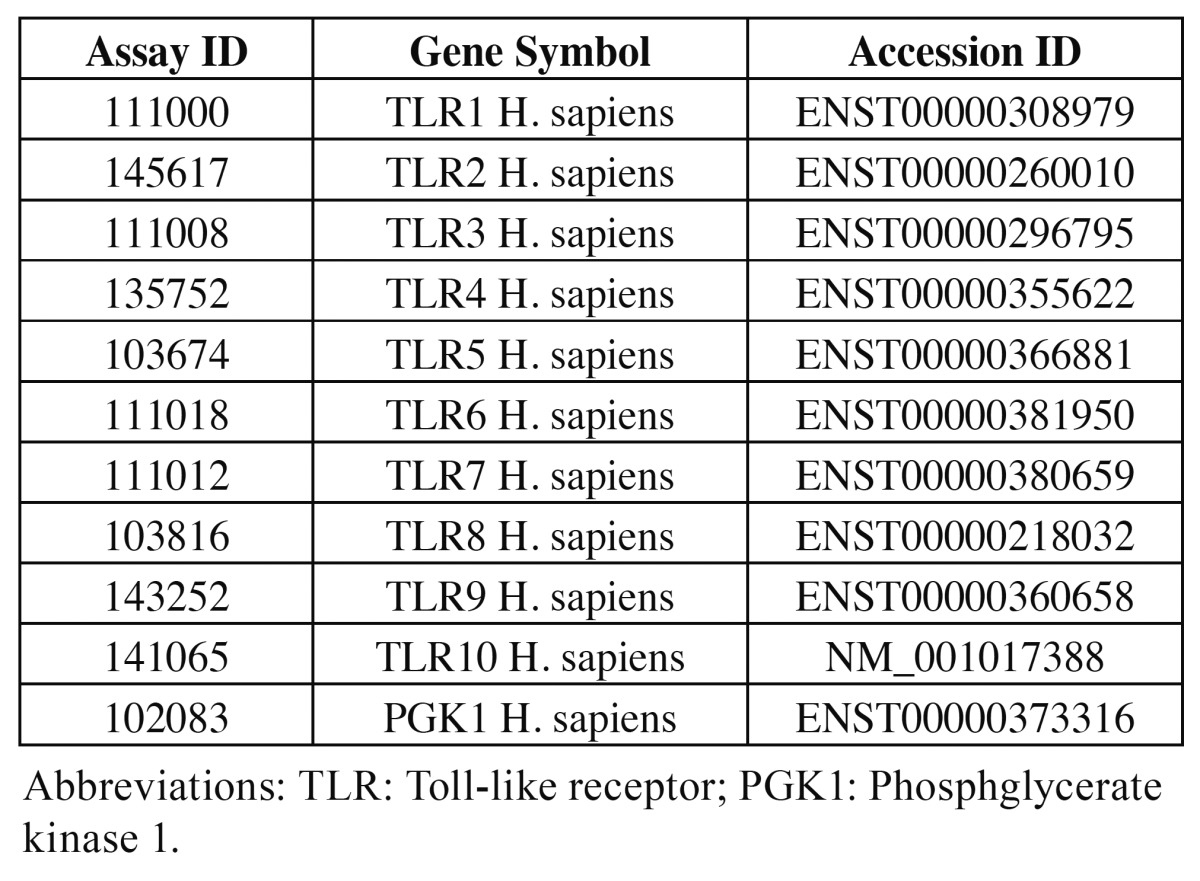
Primer names and ID used for real-time PCR (as supplied by Roche).

- Statistical analysis

The Shapiro-Wilk-Test was used to test normal distribution of the data. Differences in TLRs’ expression on m-RNA and Protein levels in G-MSCs and G-MSCs-i were evaluated using the nonparametric Wilcoxon Signed Rank test using SPSS software (SPSS version 11.5, SPSS, Chicago, IL). The level of significance was set at *p*=0.05.

## Results

- Phase contrast inverted microscopy, colony forming units and Flow cytometric analysis

**Figure 1:**
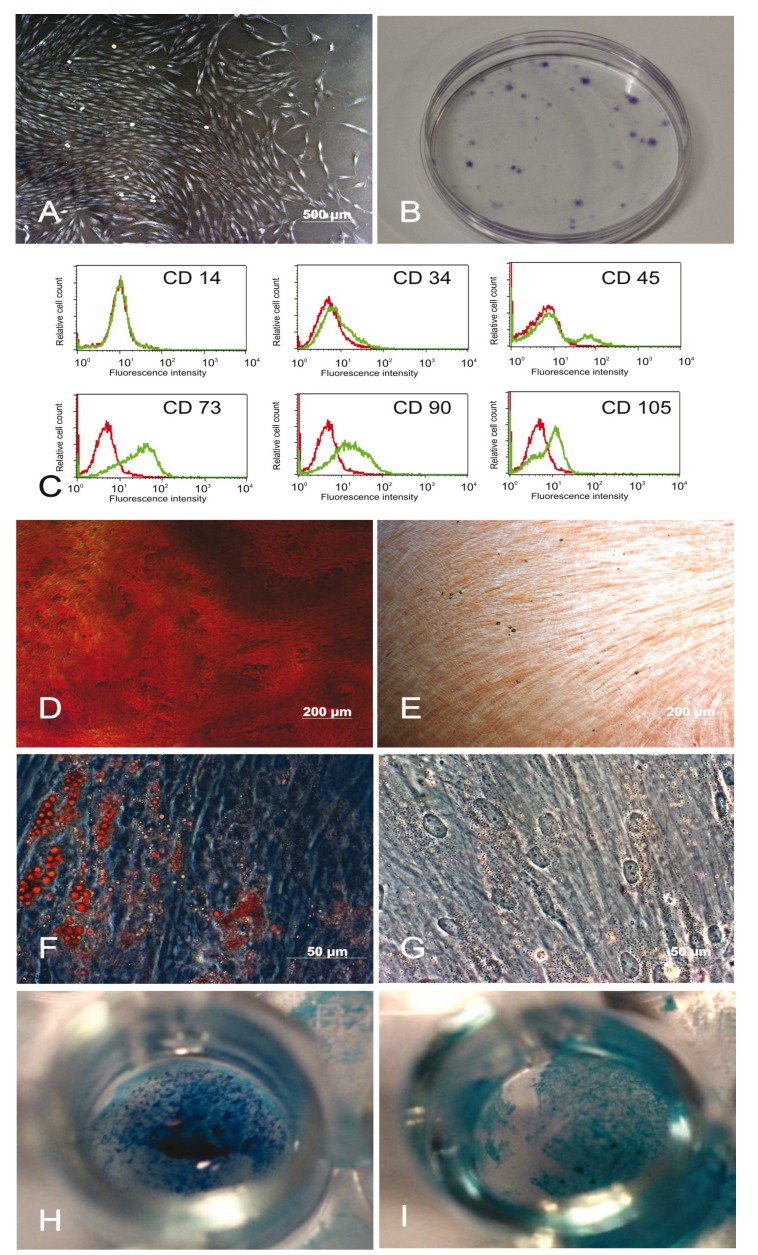
Microscopic appearance, colony-formation, surface marker expression and differentiation potential: (A) Phase contrast microscopic appearance of the adherent tissue mass with outgrowing cells (2nd week). (B) Colony-forming units of G-MSCs (crystal violet). (C) Flow cytometric analysis of the surface marker expression profile of G-MSCs. Multilineage differentiation potential of G-MSCs. Alizarin Red staining of the osteogenically stimulated G-MSCs (D) and their controls (E) Oil-Red-O staining of the adipogenically stimulated G-MSCs (F) and their controls (G). Alcian Blue staining of the chondrogenically stimulated G-MSCs (H) and their controls (I).

Following the initial adherence phase, cells grew out of the gingival tissue masses, forming adherent fibroblast-like clusters (Fig. 1A). Twelve days after seeding, G-MSCs showed CFUs (Fig. 1B). G-MSCs were negative for CD14, CD34 and CD45 while positive for CD73, CD90 and CD105 (Fig. 1C).

- Multilineage differentiation potential

Osteogenic differentiation of G-MSCs was demonstrated by the formation of calcified deposits labelled with Alizarin Red in contrast to their controls (Fig. 1D,E). Adipogenic differentiation of G-MSCs resulted in the formation of lipid droplets that were positively stained with Oil-Red-O in contrast to their controls (Fig. 1F,G). Chondrogenic differentiation of G-MSCs resulted in the formation of glycosaminoglycans positively stained with Alcian Blue in contrast to their controls (Fig. 1H,I).

- Flow cytometric TLR expression 

**Figure 2:**
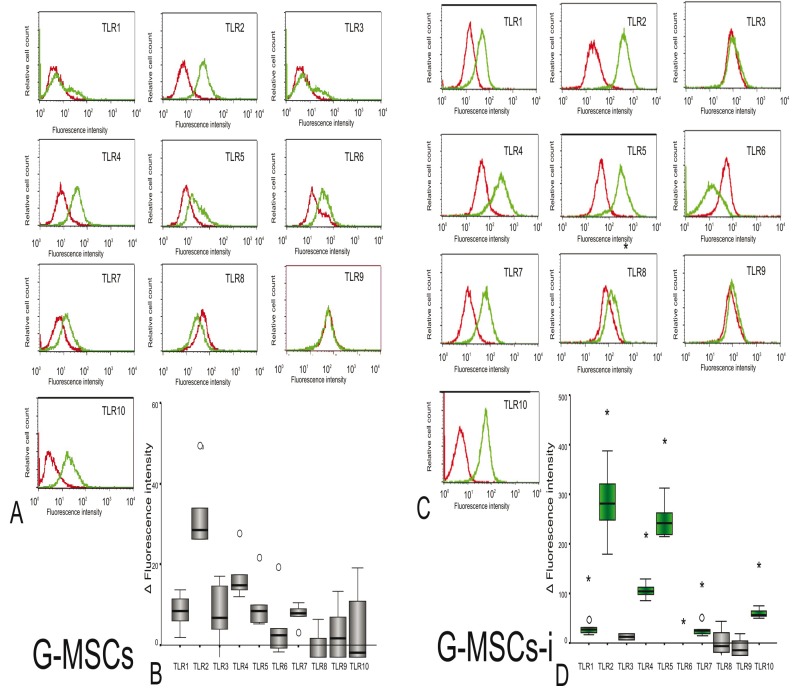
Median Flouresence Intensity (MFI) of expressed TLRs in G-MSC and G-MSC-i: (A) Median Fluorescence Intensity (MFI) of expressed TLRs 1-10 of G-MSCs (green curve) and of their isotype controls (red curve) after incubation in basic medium. (B) Protein expression of TLRs 1-10 (n=5;box- and whisker plots with medians and quartiles). (C) Median Fluorescence Intensity (MFI) of expressed TLRs 1-10 of G-MSCs (green curve) and of their isotype controls (red curve) after incubation in inflammatory medium (G-MSCs-i). (D) Protein expression of TLRs 1-10 after incubation in inflammatory medium (n=5; box- and whisker plots with medians and quartiles). The green colored boxes show increased MFI after stimulation by the inflammatory medium (Wilcoxon Signed Rank test, statistical significance marked with asterisk, *:*p*<0.05).

G-MSCs incubated in basic medium expressed (Median Fluorescence Intensity, Q25/Q75) TLR1 (8.12, 3.91/11.33), TLR2 (26.33, -2.21/41.72), TLR3 (5.79, 0.23/15.90), TLR4 (13.89, 12.92/22.63), TLR5 (7.16, 5.43/15.67), TLR6 (3.25, 0.53/11.73), TLR7 (7.83, 5.48/9.74), and TLR10 (1.16, -6.70/14.96) (Fig. 2A,B). The inflammatory medium significantly up-regulated the expression of TLR1 (25.82, 18.37/37.92, *p*=0.043), TLR2 (280.51, 213/354.90, *p*=0.043), TLR4 (105.19, 91.46/120.83, *p*=0.043), TLR5 (242.84, 216.80/287.38, *p*=0.043), TLR7 (24.41, 17.08/38.39, p=0.043) and TLR10 (55.83, 51.32/96.44, *p*=0.043), while TLR6 was no longer expressed on G-MSCs-i (-38.58, -51.64/-35.82, *p*=0.043, Wilcoxon Signed Ranks Test) (Fig. 2C,D). No difference was noted for the expression of TLR3 in both media. On the protein level, both G-MSCs and G-MSCs-i did not express TLRs 8 and 9.

- TLRs’ m-RNA expression

**Figure 3:**
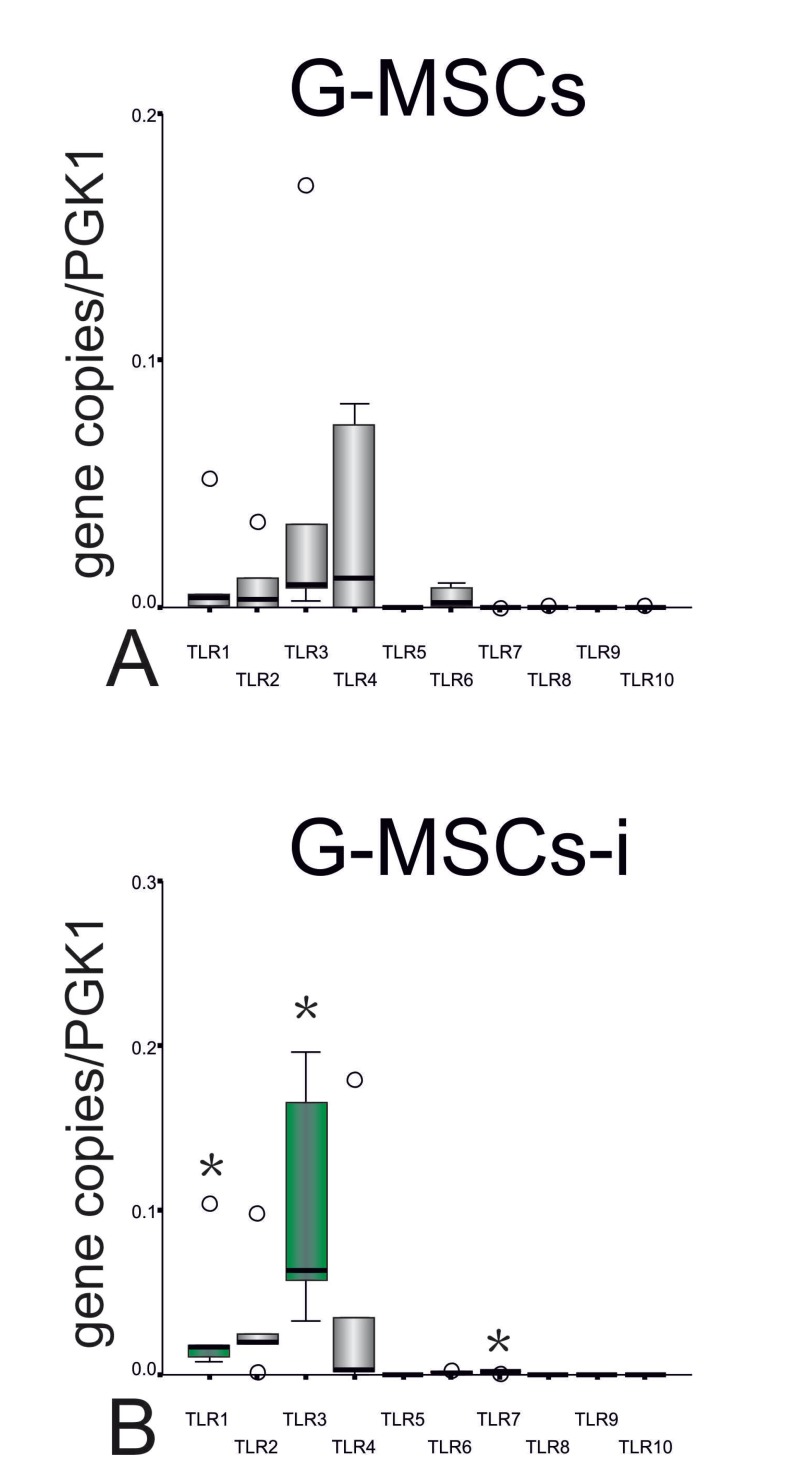
Differences in mRNA expression of TLRs 1-10 in G-MSCs and G-MSC-i: (A) m-RNA expression of TLRs 1-10 in uninflamed condition. (B) m-RNA expression of TLRs 1 to 10 in inflamed condition (n=5; box- and whisker plots with medians and quartiles). The green colored boxes show increased mRNA expression after stimulation by the inflammatory medium (Wilcoxon Signed Rank test, statistical significance marked with asterisk, *:*p*<0.05).

On the m-RNA level G-MSCs incubated in basic medium expressed (Median gene copies/PGK2copies, Q25/Q75) TLR1 (0.0037, 0.0005/0.0171), TLR2 (0.0035, 0.0002/0.0175), TLR3 (0.0091, 0.0065/0.0678), TLR4 (0.0118, 0.0000/0.0760), TLR5 (0.0001, 0.0000/0.0001), TLR6 (0.0018, 0.0004/0.0085), TLR7 (0.0000, 0.0000/0.0001),TLR8 (0.0000, 0.0000/0.0003) and TLR10 (0.0000, 0.0000/0.0002) (Fig. 3A). G-MSCs-i showed a significantly higher expression of TLR1 (0.0172, 0.0094/0.0611, *p*=0.043), TLR3 (0.0629, 0.0449/0.1805, *p*=0.043) and TLR7 (0.0015, 0.0011/0.0022, *p*=0.043, Wilcoxon Signed Ranks Test) in addition to downregulated TLR6 and TLR10 (Fig. 3B).

## Discussion

In the field of tissue engineering, therapeutic approaches may employ direct G-MSCs’ transplantation into inflamed environment (13,15), resulting ultimately in a direct interaction between the G-MSCs and the PAMPs through their TLRs. The aim of the present study was to characterize for the first time the distinctive TLRs’ expression profile of G-MSCs in inflamed as well as in uninflamed conditions as a first stage of exploring this possible interaction.

Similar to previous studies (15,27) the putative stem cell marker, STRO-1, implemented to isolate and purify bone marrow stromal stem cells (BMSSCs) (33) and alveolar bone proper-derived stem/progenitor cells (34) using immunomagnetic cell selection has been exploited to isolate the G-MSCs. The characterized G-MSCs showed all classical features defined for MSCs (30,35), being positive for CD73, CD90 and CD105, while negative for CD14, CD45 and CD45, as well as demonstrating a remarkable CFUs ability, plastic adherence and a multi lineage differentiation potential, into osteogenic, adipogenic and chondrogenic directions.

The development of an inflammation follows most tissue injuries, being an integral part of the early healing process. Transplanted MSCs are exposed to such stimuli in many clinical and therapeutic conditions. Even under clinically healthy conditions or after a successful anti-inflammatory periodontal therapy, the periodontal tissues show histologically pre-inflammatory conditions (36). Previous studies demonstrated MSCs’ sensitivity to inflammation (24,32,37,38). In the current study, G-MSCs were cultured in a medium supplemented with IL-1β, IFN- γ, TNF-α, and IFN-α, the cytokines mostly present at inflammatory sites (24) as well as in basic medium.

On protein level, the G-MSCs in uninflamed condition showed a distinctive expression profile of TLRs 1, 2, 3, 4, 5, 6, 7 and 10 in different quantities without TLR8 and TLR9 expression. According to their median expression values, TLR2 was highest expressed followed by TLRs 4, 1, 7, 5, 3, 6 and finally 10. The inflammatory medium significantly up-regulated the expression of TLRs 1, 2, 4, 5, 7, and 10 and diminished TLR6 expression in G-MSC-i. It further partially altered the quantitative order of expression with TLR2 remaining highest expressed followed by TLRs 5, 4, 10, 1, 7 and finally 3. The m-RNA level of most G-MSCs’ TLRs in inflamed and uninflamed conditions correlated with the protein expression, with a statistically significant upregulation reached for TLRs 1, 3 and 7 and a down regulation in TLR6.

Contrasting TLRs expression profiles have been reported on human MSCs originating from different tissues. TLRs 1, 2, 3, 4, 5, 6 and 9 were expressed in umbilical cord blood MSCs (39,40). Bone marrow derived MSCs showed a broader expression pattern with added TLRs 8 and 10 expression (41-43). Wharton jelly’s MSCs demonstrated a comparable pattern with marginal or deficient expression of TLR4 (41,44). Studies on dental tissue derived MSCs recorded the expression of TLRs 2, 3 and 4 in dental follicle MSCs (45,46) and dental pulp MSCs (45,47). TLRs 1, 2, 3, 4, 5, 6, 8, 9 and 10 were expressed in periodontal ligament MSCs (43). Similar to our results inflammation tended to upregulate the expression of TLR2 (42,48), TLR4 (42,49) and TLR7 (48) as well as to downregulate the expression of TLR6 (42) in BM-MSCs.

The currently outlined TLR expression profile, especially under inflammation, may influence the G-MSCs’ therapeutic potential *in-vivo*. An upregulation of the LPS sensing TLRs 2 (50,51) and 4 (51,52) could increase the G-MSCs’ ability to recognize gram-negative periodontal pathogens, including Aggregatibacter actinomycetemcomitans, Porphyromonas gingivalis, Prevotella intermedia and Tannerella forsythia. The inflammatory upregulation of TLR5 expression could also favor the recognition of bacterial flagellin of periodontal pathogens (52,53) as Treponema denticola (54,55), Campylobacter rectus (55,56) and Eubacterium species (55,57). TLR1 and TLR2 upregulation could favor lipoproteins’ recognition (55), while an upregulated TLR7 and TLR9 will heighten the ability to recognize viral pathogens (58,59).

The current study describes for the first time the distinctive TLRs’ expression profile of G-MSCs in inflamed and uninflamed conditions, which could impact its therapeutic potential in inflammatory environments *in-vivo* (26). In light of the present results, inflammation tends to upregulate most TLRs’ expression, promoting the ability of G-MSCs to recognize important periodontal PAMP *in-vivo*.
